# Integrating remote international experience and community engagement into course‐based animal behavior research

**DOI:** 10.1002/ece3.9721

**Published:** 2023-01-10

**Authors:** Adam D. Kay, Zach Lager, Litha Bhebheza, Justa L. Heinen‐Kay

**Affiliations:** ^1^ Biology Department University of St. Thomas St. Paul Minnesota USA; ^2^ Sibanye South Africa Stellenbosch South Africa; ^3^ Natural Sciences Department Metropolitan State University St. Paul Minnesota USA

**Keywords:** course‐based undergraduate research (CURE), goats, South Africa, STEM education, video‐based research

## Abstract

Human‐centered, active‐learning approaches can help students develop core competencies in biology and other STEM fields, including the ability to conduct research, use quantitative reasoning, communicate across disciplinary boundaries, and connect science education to pressing social and environmental challenges. Promising approaches for incorporating active learning into biology courses include the use of course‐based research, community engagement, and international experiences. Disruption to higher education due to the COVID‐19 pandemic made each of these approaches more challenging or impossible to execute. Here, we describe a scalable course‐based undergraduate research experience (CURE) for an animal behavior course that integrates research and community engagement in a remote international experience. Students in courses at two U.S. universities worked with community partners to analyze the behavior of African goats grazing near informal settlements in Western Cape, South Africa. Partners established a relationship with goat herders, and then created 2‐min videos of individual goats that differed in criteria (goat sex and time of day) specified by students. Students worked in small groups to choose dependent variables, and then compared goat behavior across criteria using a factorial design. In postcourse surveys, students from both universities indicated overall enthusiasm for the experience. In general, students indicated that the laboratory provided them with “somewhat more” of a research‐based experience compared with biology laboratories they had taken of similar length, and “somewhat more” to “much more” of a community‐engagement and international experience. Educational benefits were complemented by the fact that international educational partners facing economic hardship due to the pandemic received payment for services. Future iterations of the CURE can focus on goat behavior differences across ecological conditions to help herders increase production in the face of continued environmental and social challenges. More generally, applying the structure of this CURE could facilitate mutually beneficial collaborations with residents of under‐resourced areas around the world.

## Graphical abstract

Course‐based research, community engagement, and international experiences can enhance undergraduate biology education, but each were made more challenging due to the COVID‐19 pandemic. Here, we describe a scalable course‐based undergraduate research experience focusing on African goat behavior that integrates research and community engagement in a remote international experience. Educational benefits for students were complemented by the fact that international educational partners facing economic hardship due to the pandemic could receive payment for services.

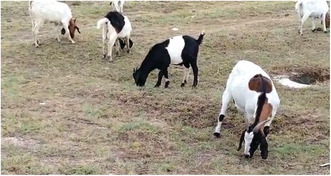

## 1 INTRODUCTION

Over the last few decades, numerous publications have helped reimagine undergraduate biology education in a way that is more focused on students (Laursen, 2019). A key contribution to this development, the 2011 Vision and Change (V&C) document, identifies core competencies that all students should develop, including the ability to apply the process of science, to use quantitative reasoning, to communicate across disciplinary boundaries, and to explore science in a social context (AAAS, 2011). Human‐centered, active‐learning approaches can help students develop these competencies in part by increasing their interest in and level of engagement with course content. This increased engagement can narrow achievement gaps (Theobald et al., 2020), and reduce attrition from STEM majors, particularly for underrepresented students (Freeman et al., 2014; Haak et al., 2011).

One main approach for realizing V&C recommendations is through research‐based instruction. Developing opportunities for authentic research experiences for undergraduates is essential for capturing the imagination of students by exposing them to the creativity of STEM disciplines (PCAST, 2012). One approach for broadening access to research opportunities is through the development of course‐based research experiences (CUREs), which have shown generally to increase undergraduate engagement in research and to broaden participation (Bangera & Brownell, 2014; Rodenbusch et al., 2016; Stanford et al., 2017). One reason why CUREs have been successful in providing opportunities to more students is because, unlike research internships or apprenticeships, they are often less costly for institutions and do not require time or work commitments from students that exceed course requirements (Bhattacharyya et al., 2020).

A second promising approach for creating active‐learning experiences is through community‐engaged education (Enos, 2015). Community‐based learning is usually organized around projects with public benefit that are done in collaboration with community partners. Community‐engaged education has received more attention due to the growing emphasis on producing graduates who have sufficient empathy and understanding to address pressing social challenges (Hansen et al., 2021). Community‐oriented experiences can increase student engagement and student learning connected to course content (Ryan, 2017; Tannenbaum & Berrett, 2005) due at least in part to the fact that students are highly motivated by opportunities to help others (Gorski et al., 2015). Despite its potential benefits, incorporation of community‐engaged learning into U.S. undergraduate biology education is rare (Kay et al., 2022; Zizka et al., 2021). Barriers to broader implementation include resources, time, or experience to develop connections with community partners, and challenges in obtaining institutional support (Mehta et al., 2015).

A third approach for creating active‐learning experiences for biology students is through international education (Coker et al., 2018; Hope, 2008; McLaughlin, 2021). Intentional international experiences can provide students with a range of benefits including improved ability to put learning in context, development of interpersonal skills, and enhanced collaboration (Coker et al., 2018). International experiences in biology and other STEM disciplines are often oriented around research experiences and occasionally involve community‐engaged learning.

COVID‐19 disrupted the entire landscape of higher education but had a particularly strong impact on international education experiences (McLaughlin, 2021) as universities reduced or completely restricted study abroad programs. In response, there was increased emphasis placed on developing remote experiences that could help students make some connection to distinct cultures (Liu & Shirley, 2021). Such modifications may have to become more common in the future if pandemics, strife, high costs, and ecological considerations make international travel less appealing to students and program managers. In the face of such restrictions, what is needed are programs that can help to broaden student perspectives and connect them to others without having to travel.

Here, we describe a CURE for a biology course that incorporates community‐based learning with an international partner, designed specifically to bring together many of the high‐impact educational practices identified by the American Association of Colleges and Universities (Kuh & O'Donnell, 2014). Developed for an animal behavior‐type course, the CURE focuses on student analysis of brief videos of domestic goats grazing near informal urban settlements in western South Africa. The videos were created in partnership with community members who were paid for their work from course budgets. Below, we describe the development of the CURE, results from its implementation, and ideas for how it could evolve in future iterations.

## 2 STUDY ORGANISM AND COMMUNITY CONTEXT

Course‐based undergraduate research experiences are defined as authentic research experiences that involve whole classes of students and that aim to address a question of interest to the scientific or general community (Auchincloss et al., 2014). Although many types of experiences are presented as CUREs, we sought to stay true to the above definition by emphasizing the “authentic” aspect of a CURE (by giving students some control over experimental design) and the “question of interest” aspect (by envisioning how results could be useful to community partners).

**FIGURE 1:**
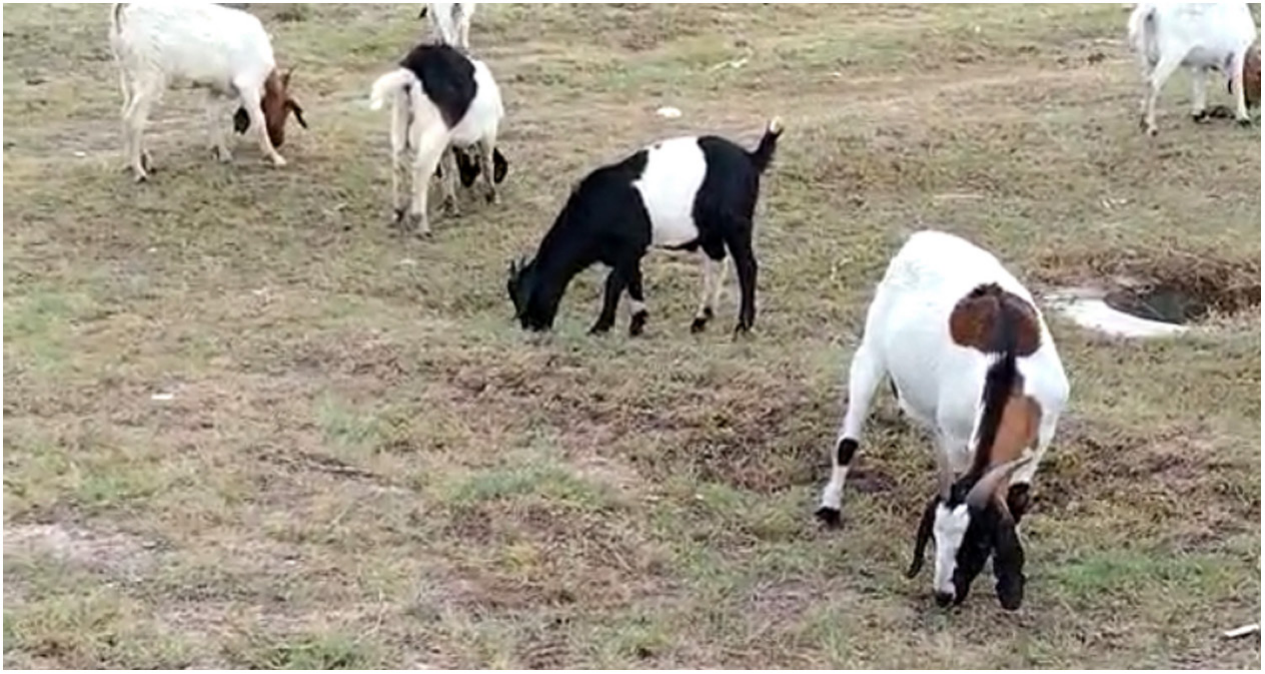
Indigenous Nguni goats grazing near an informal settlement around Klapmuts, Western Cape Province, South Africa.

The CURE we developed is oriented around videos of indigenous Nguni goats grazing near an informal settlement in the Western Cape Province, South Africa (Figure 1). Goats are an important livestock animal in many developing countries (Simela & Merkel, 2008), providing meat, milk, and hides for home consumption and exchange (Peacock, 2005).

Goats play a particularly important role in domestic affairs in much of Africa. There are approximately 425 million goats in Africa, with roughly 5.2 million of these in South Africa (Mataveia et al., 2021). In South Africa, there are both a commercialized, intensive goat production system, and a semi‐intensive system that consists of small farming cooperatives and individual farmers (Mdladla et al., 2017; Morrison, 2007). The Nguni goats used in this study are part of this semi‐intensive “village” goat system, which is characterized by animals with limited housing and veterinary care that are generally adapted to harsh conditions. This production system usually consists of informal labor, with herders overseeing small herds with minimal use of technology and other inputs (Mataveia et al., 2021). Production performance and goat behavior in these village goat systems are much less studied than in commercialized systems (Masika & Mafu, 2004; Ncube et al., 2020), but research on these systems could help contribute to management practices that provide benefits to marginalized communities.

Goats observed in this study were housed and grazed near informal settlements near Klapmuts in Western Cape Province, South Africa. These informal settlements are part of a sprawling network of over 450 old apartheid townships, housing projects, shack communities, and temporary structures across the Greater Cape Town area (Amin & Cirolia, 2018). Many of these settlements lack some if not all basic amenities (e.g., municipal water or sewage, electricity). Goats around these settlements likely exclusively rely on natural, often highly disturbed pastures in nearby peri‐urban areas. Goats usually graze during daylight hours and then are returned to their paddocks. The goats used in this study were part of a herd of about 60 animals. They were not tethered, and often grazed as part of a mixed herd with cattle.

## 3 BASIC DESIGN AND DEVELOPMENT OF THE GOAT CURE

We initiated our Goat CURE after the COVID‐19‐related cancelation of a biology study abroad course, “Urban Agriculture and Social Innovation,” which was scheduled to go to Cape Town, South Africa, in June 2020. In response to the cancelation, we sought to create a CURE in a Spring 2021 Biology course (Animal Behavior) at the University of St. Thomas (MN, “St. Thomas”) that benefited students but also provided income for people in Cape Town facing economic hardship. This CURE was then replicated in a Behavioral Ecology course at Metropolitan State University (MN, “Metro State”) in Summer 2021.

**FIGURE 2:**
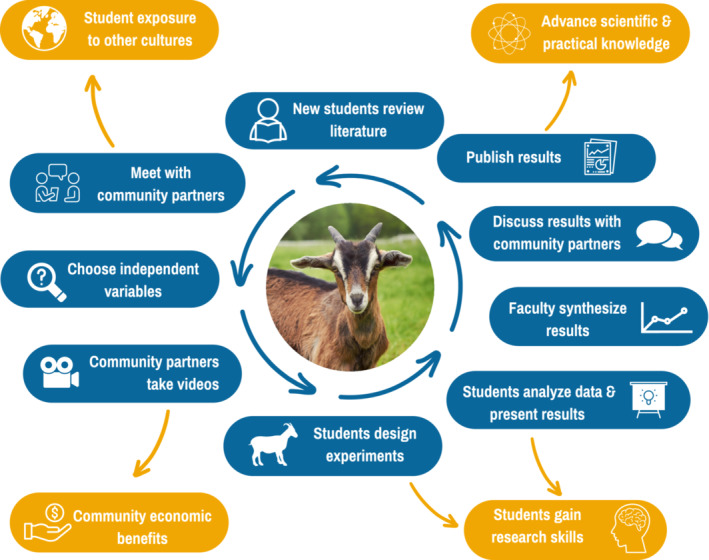
Idealized framework for the Goat CURE. Blue bubbles indicate structural components of the CURE and orange bubbles describe outcomes. Starting from top left, new students start by *reviewing literature* and other materials, then *meet with community partners* to learn more about them and the context. Students, faculty, and partners then *choose independent variables* (e.g., time of day) around which videos should be structured. After *partners take videos*, students in small groups view videos and *design their own experiment* by deciding on dependent variables (e.g., time spent foraging). Students then *analyze their data* and then *faculty synthesize results* from all groups and *discuss those results with community partners*. Ideally, *results can be published* and inform a subsequent group of students in the CURE. Main outcomes are *student exposure to other cultures*, *community economic benefits*, *student acquisition of research skills*, and *advancement of practical knowledge*. See text for more detail.

The design of the Goat CURE is described in Figure 2, and its implementation schedule is presented in Table 1. Prior to the start of the first laboratory, the community partners (authors LB and ZL) initiated contact with herders managing goats near Klapmuts. Urban and peri‐urban goats had been a topic of previous conversations among all coauthors because of the authors' shared interests in urban agriculture and township economic development. LB and ZL became aware of the specific herds near Klapmuts because LB had seen them regularly when traveling to work near Klapmuts. To initiate contact with herders, LB approached the informal settlement outside of which goats were penned overnight and asked residents for help finding the herders. After making contact, LB spent time gaining the trust of the herders, who were apprehensive about possible theft. After negotiating payment for the herder, LB and ZL took initial panoramic videos of the herd to provide the U.S. students with a sense of context.

**TABLE 1:** Schedule for how the Goat CURE was implemented at each university

Laboratory period	Activity
Before period 1	Community partners take panoramic videos of goat herds
1	Students watch cultural videos and conduct literature view about goat behavior. Students develop goat‐related questions for community partners
2	Students meet (remotely) with community partners to learn about their histories and the system, and to ask questions. Students decide on independent variables (e.g., goat sex and time of day) around which to structure videos
Between periods 2 and 3	Community partners create videos
3	Students use videos to test a hypothesis that they develop
4	Students analyze results and prepare a presentation
After period 4	Faculty synthesize results and share with community partners. Data can be prepared for external audiences (presentations and publications) and future possibilities are discussed

In an initial laboratory period (Week 1), students viewed the panoramic videos and conducted a literature search to learn more about African goats and goat behavior (upper left in Figure 2). Students were also asked to watch short cultural videos explaining the causes and consequences of informal settlements in South Africa. They were then asked to develop a series of questions to ask the community partners (LB and ZL) and the goat herders. These questions were a mix of ecological questions (“What is the social structure/hierarchy of the goat herd? What is the general risk of predation?”), social (“What are the goats used for? How much do goat herders get paid?”), and practical (“How much time might you be able to spend filming the goats?”). The class then held a zoom session with LB and ZL during a subsequent lecture period. Before the session, students were informed that the community partners were friends of the instructors (ADK and JHK) and were eager to answer any of their questions. During this session, LB and ZL introduced themselves, recounted their personal histories, and answered questions. They also submitted written answers to the students' questions.

In Week 2, students were organized in small groups (3–4) and tasked with determining what might serve as independent variables that could facilitate hypothesis testing using short videos of individual goats. These suggestions were then filtered by all of the authors, who ultimately decided to create videos that would allow students to contrast goat sex (male vs. female) and time of day (morning vs. late afternoon).

Between weeks 2 and 3, LB conducted all filming over the course of 1 day. Each video was ~2 min and focused on one individual (see Video S1). LB created 32 videos in total—16 in the morning and 16 in late afternoon, half of which in each session were of male goats and half were of females.

In Week 3 of the laboratory, students were asked to design an experiment and test a hypothesis of their choice using these videos. First, student groups were asked to view several videos and then decide as a group on the dependent variable(s) they would focus on. They were informed that variables would have to be something they could quantify using only observations of the videos. Examples of dependent variables chosen by students included % time spent feeding, % time spent scanning, and % time with tail raised. Then, each group of students watched all 32 videos and quantified their chosen variable(s). Students were asked to have each video independently evaluated by two group members to test for possible observer effects.

In Week 4, students analyzed their results using statistical training from previous laboratory periods. After analysis, each group made a short oral presentation to their peers and prepared a final written report to be shared with the community partners.

The educational and community outcomes we envisioned for this CURE are also presented in Figure 1 (orange bubbles). First, we wanted to create paid work for community partners. Coauthors and goat herders were paid for developing the project and for the creation of videos using course budgets that would otherwise have been used for laboratory supplies. Further information about this economic exchange is available from the corresponding author upon request. Second, we sought to help students develop research skills by asking them to conduct a guided, start‐to‐finish experiment in the context of a laboratory class. Third, we wanted to expose students to another culture and allow them to interact collaboratively with community partners. Finally, we wanted to have course‐based research contribute to the general scientific community through presentations and publications. Although this final outcome has not been realized, we hope that future iterations will allow for publication of results that can contribute to knowledge about this under‐researched system. Outcomes and future opportunities are described more thoroughly below.

## 4 STUDENT IMPRESSIONS OF THE CURE

We surveyed students from St. Thomas and Metro State in December 2021, 7 and 4 months, respectively, after the courses were completed. All survey work was first approved by the Institutional Review Boards at St. Thomas and Metro State. Student participation in the survey was voluntary, and no individual identifiers were collected. We sent the survey to the 47 students who had taken either course and received 10 responses from St. Thomas and nine responses from Metro State students (overall response rate = 40%). Students received no compensation for responding.

We asked students seven multiple‐choice questions and four written‐response questions. The multiple‐choice questions asked students to compare their experience in the African Goat laboratory to “labs of similar length that you have had in other biology classes” (Table 2). Answer options for these questions ranged from “much less” to “much more” on a 5‐point scale. Questions focused either on the research aspect, the community engagement aspect, or the international aspect of the laboratory. The written‐response questions asked about the strengths of the course, the strengths of the laboratory, and any thoughts about how the laboratory could be improved in the future. We also included a question that gave students an opportunity to share other thoughts about the laboratory or the course.

**TABLE 2:** Quantitative student feedback about the Goat CURE

Question	Response (mean + 1 SE) (range: 1 = much less, 5 = much more)
Research‐oriented questions	
Compared to laboratories of similar length that you have had in other biology classes, to what extent did this experience allow you to develop your own hypothesis?	4.105 ± 0.201
Compared to laboratories of similar length that you have had in other biology classes, to what extent did this experience allow you to develop your own methods for collecting data?	3.895 ± 0.264
Compared to laboratories of similar length that you have had in other biology classes, to what extent did this experience ask you to analyze and interpret your own data?	4.158 ± 0.191
Community engagement‐oriented questions	
Compared to laboratories of similar length that you have had in other biology classes, to what extent did this experience allow you to interact with members of a community that was different from your own?	4.474 ± 0.193
Compared to laboratories of similar length that you have had in other biology classes, to what extent did this experience help you connect societal challenges to your biology education?	4.421 ± 0.159
International experience‐oriented questions	
Compared to laboratories of similar length that you have had in other biology classes, to what extent did this experience allow you to gain insight about another part of the world?	4.632 ± 0.114
Compared to laboratories of similar length that you have had in other biology classes, to what extent did this experience allow you to gain insight into another culture?	4.526 ± 0.118

Multiple‐choice responses suggest that, overall, students valued the research, community engagement, and international aspects of the laboratory (Table 2). Mean responses to all questions were much more positive than the option of being “about the same” as other laboratories (mean ± SE = 4.31 ± 0.07, where 3—about the same, 4—somewhat more, and 5—much more). Average responses to research component questions that asked whether the laboratory allowed students to develop hypotheses, develop data collection methods, and analyze and interpret their own data more than similar laboratories ranged from ~3.9 to 4.1, and average responses to community engagement questions and international experience questions ranged from 4.4 to 4.6. Differences in responses among question types (research, community, and international) were almost significant (*F*_2,54_ = 3.076, *p* = .0543).

Written response questions showed that students identified and valued the learning objective targets but struggled somewhat with the open‐ended nature of the laboratory (Selected answers presented in Table 3). When asked “In general, what did you think were the strengths of this lab?,” seven students mentioned the international aspect, five mentioned the value of the research‐based inquiry, and two mentioned community engagement or service; five other responses praised the laboratories' organization and execution. When asked “How do you think this lab could be improved in the future?,” five students mentioned that they would have liked the opportunity to make more comparisons (e.g., across seasons), and five mentioned that they would have liked to have had more specific instructions about what to do (e.g., instructors should identify which behaviors to study). In addition, two students mentioned logistical issues (problem with laboratory partners and the online format), and six students either did not answer the question or answered that they had no suggestions for improvement. When asked “Is there anything else you would like to say about this lab?,” 12 of 19 students provided answers. Four of the answers mentioned enthusiasm for the international component, two mentioned enthusiasm for the community engagement, five expressed general enthusiasm for the laboratory, and two mentioned the lack of specific instructions.

**TABLE 3:** Qualitative student feedback about the Goat CURE

Question	Response	Categorization
1. Strengths of laboratory:	“I thought this was an incredibly unique lab experience, no other classes have had an opportunity like it. We really were able to develop our own research in ways that other labs cannot.”	Research‐based inquiry
	“Connection to other countries, Understanding new ways of life/culture”	International experience
2. Aspects to improve:	“A more varied clip set, other aspects of goat behavior to focus on. Longer term perhaps seasonal variations could be interesting. Interviews with farmers to get a wider range of perspective.”	Opportunities for more comparisons
	“Maybe have a specific thing to look for about the goats”	Process uncertainty
3. Other comments:	“It opened my eyes, connecting society and biology.”	Community engagement
	“While it ended up great, the process of getting there was a little confusing”	Process uncertainty

There are several limitations to our survey data, and thus our assessment of this laboratory should be considered preliminary. First, only a fraction of students responded to the survey, and respondents may have been particularly enthusiastic about the laboratory, the course, or their education in general. Second, we lacked true controls (e.g., similar students in a similar course that did not use this laboratory). We adjusted to this lack of a true control by asking students to compare their experiences to those in other biology laboratories. Third, survey questions asked students to compare this experience to their previous experiences rather than an objective standard. As a result, survey responses may differ if the same laboratory is taught by different instructors or at other institutions with more (or fewer) research, community engagement, or international experiences incorporated into the curriculum. Fourth, our survey relies on students' opinions rather than learning outcomes. However, our main objective was to increase students' engagement, so the enthusiasm expressed in their responses provided a reasonable measure of whether we achieved this objective. Finally, our survey was administered several months after the experience. Regardless of these limitations, our survey responses suggest that at least respondents viewed the laboratory as offering a “somewhat” to “much” better research, community engagement, and international experience compared to alternative opportunities in their biology programs.

## 5 POSSIBILITIES FOR ITERATIONS AND EXPANSION

A main value of CUREs is the possibility of engaging many students in relevant research that is iterative. Video‐based CUREs focused on the behavior of goats or other domesticated animals are ideally suited for iteration given the relative ease with which short videos of individual animals can be taken. The possibility of iteration as described in this Goat CURE can also build and reinforce relationships with individuals and communities that are remote from one another.

Several students' comments suggested opportunities for iteration focused on additional temporal comparisons (e.g., across seasons). Such comparisons have been made previously in direct observation studies. For example, Bakare and Chimonyo (2011) found that three goat genotypes (Xhosa lop‐eared, Nguni, and Nguni × Boer crossbred) spent similar time foraging across seasons in a semi‐arid rangeland in South Africa. Information from additional video projects as described here could help to broaden and generalize this finding. Moreover, other behaviors such as grooming, tail positioning, general movement, and vocalization could all be explored across seasons, in addition to the sex and time‐of‐day comparisons made in the initial version of this CURE.

Other iterations are possible by creating videos that allow for local or regional spatial comparisons. Examples include creating opportunities for students to assess behavioral differences across habitats or rangeland quality. Snapshot samples of forage conditions could also allow students to assess some ecological variables (e.g., % cover and plant diversity). Community partners could be hired to make some of these ecological measurements, creating additional employment opportunities.

A critical opportunity for iteration is to create extended dialogue among students, community partners, and community members (including goat herders). Successive iterations of the Goat CURE could allow for students to share findings with herders, who then could provide feedback on whether students' observations were consistent with herder knowledge and practices. Such exchange could deepen students' understanding of goat behavior and help them see the useful but potentially incomplete knowledge that comes from scientific studies.

One other opportunity for improvement that emerged from students' comments is students' apparent need for more explicit instruction. Several students mentioned that they would have liked more guidance about which behaviors to study and exactly how they should quantify them. Given that a main value of CUREs is to provide students with an opportunity for self‐directed, original discovery, this lack of guidance is actually an essential part of the experience. What we learned from this experience is that we need to communicate more effectively about the CURE's objectives and their rationale, and the general value of CUREs for students. The implementation of CUREs in more courses, especially early in the curriculum, should also help in making students more comfortable with the open‐ended nature of the scientific process.

## 6 REMOTE VIDEO CURES TO CONNECT BIOLOGY EDUCATION TO GLOBAL CHALLENGES

Our analysis of the Goat CURE suggests standardized video segments taken by international community partners can provide substrate for original student‐led research while exposing students to distant social and environmental challenges. From student surveys and community partner testimonials, we can point to advantages of this approach and opportunities for improvement and expansion.

Our work builds upon previous published activities that have incorporated video footage into animal behavior research and teaching (Bain et al., 2021; Grigg et al., 2021; Littman & Moore, 2016; Nunes et al., 2021; Zuerl et al., 2022). Previous studies have incorporated videos from camera traps in the field (Burger et al., 2021) or in zoos (Hahn‐Klimroth et al., 2021), citizen science contributions (Oberbauer et al., 2021), or targeted footage of wild (Bain et al., 2021), domesticated (Grigg et al., 2021; Vega et al., 2010), or laboratory animals (Kimura et al., 2014). The use of video footage in animal behavior can help reduce observer effects and allow for the application of standardized algorithms for behavioral analysis, including machine learning. As an education tool in animal behavior, video footage can expose many students to field conditions (Littman & Moore, 2016). Although remote, video‐based education will never capture the many benefits of direct exposure to field conditions, the salient question is whether such experiences are an improvement over typical laboratories at home institutions. Here, we build on this previous work by developing video‐based work in an animal behavior course that helps students simultaneously conduct original research, develop community connections, and gain international experience.

The first goal of this work was to help students participate in course‐based original research, characterized by five features: use of scientific practice, collaboration, discovery, iteration, and the opportunity to produce useful knowledge (Auchincloss et al., 2014). *Use of scientific practice*, from question generation through the analysis and presentation of results, is difficult to achieve in course settings due to time and other logistical constraints. Our use of short videos of individual animals in the field, taken in a way to make specific comparisons (across sexes and times of day), provides an efficient way for students to ask distinct research questions on dependent variables of their choice. Although this approach obviously does not include every aspect of the scientific process (e.g., identifying a study system), it provides students with significant opportunity for asking their own questions. The Goat CURE also provides clear opportunities for *collaboration* and *discovery* given that students worked in small groups and chose their own dependent variables to study original video segments. Collaboration opportunities also existed through repeated interactions with community partners. *Iteration* opportunities exist as described above. Future iterations may help to identify goat behavior differences across time and space that can help researchers understand constraints on African goat rearing in the understudied, semi‐intensive production system, and potentially provide herders with information about herd condition. These broader comparisons can increase the extent to which this CURE gives students the *opportunity to produce useful knowledge*. Overall, students indicated that they viewed the Goat CURE to have “somewhat more” opportunity to conduct original research (i.e., develop hypotheses, develop data collection methods, and analyze and interpret their own data) relative to “labs of similar length that you have had in other biology classes.” Additional footage across different conditions will likely enhance students' impressions that they are making important contributions to an original research effort.

Another main goal of this work was to create community engagement opportunities for students. Community‐based learning experiences are rare in U.S. undergraduate biology courses (Kay et al., 2022) even though such experiences can help students develop empathy and connect their education to pressing environmental and social challenges. Students in both surveyed programs indicated that they thought that the Goat CURE allowed them “to interact with members of a community that was different from your own” and “connect societal challenges to your biology education” somewhat more to much more than did their other biology courses (Table 1). In comments, a student wrote how the experience “…opened my eyes, connecting society and biology,” another wrote how this “unique community partners experience … opened us up to a [broader] perspective…,” and a third wrote that they most valued how the laboratory “benefitted study here and livelihoods there.” While community engagement would be strengthened by extended, in‐person interactions, our experience with this laboratory suggests that the combination of cultural instruction, open‐ended video discussions with partners, and start‐to‐finish research opportunities for students does enough for students for them to feel like their course experience is significantly more connected to broader societal challenges than experiences in similar courses. Increasing such opportunities in STEM has significant potential to diversity STEM fields by increasing retention of underrepresented minority students, first‐generation students, and communally oriented men and women (Boucher et al., 2017).

The other main goal of this work was to create an international experience for students given the travel restrictions created by the COVID‐19 pandemic. Students in both surveyed programs thought that the Goat CURE allowed them to gain insight about “another part of the world” and “another culture” “somewhat more” to “much more” than did their other biology courses (Table 1). Several students mentioned that they viewed the laboratories' main strength as its ability to provide students with an opportunity to “study something across the world” and to “connect… to other countries [and] understand… new ways of life/culture.” Future work should explore the extent to which this type of experience can replicate some of the educational benefits associated with international experiences (e.g., connecting learning to global issues, developing interpersonal skills, and collaboration skills) relative to traditional on‐campus experiences.

The COVID‐19 pandemic has been disruptive to higher education but has also created opportunities for innovation. Given ecological, financial, and other potentially long‐lasting constraints on international travel, new approaches are needed for broadening students' perspectives and for connecting course content to societal issues. Our work suggests that personally tailored videos of animal behavior, created by community partners in distant locations, can provide novel research opportunities for students while familiarizing them with social and environmental challenges in places that are unfamiliar to them. Scaling up such projects will allow for deeper assessment of student benefits and provide additional financial support to community partners in lower income communities around the world.

## AUTHOR CONTRIBUTIONS

**Adam D. Kay:** Conceptualization (equal); formal analysis (equal); funding acquisition (lead); methodology (equal); project administration (equal); writing – original draft (lead); writing – review and editing (equal). **Zach Lager:** Conceptualization (equal); investigation (equal); methodology (equal); project administration (equal); writing – original draft (supporting); writing – review and editing (equal). **Litha Bhebheza:** Conceptualization (equal); methodology (equal); project administration (equal); writing – review and editing (equal). **Justa L. Heinen‐Kay:** Conceptualization (equal); formal analysis (equal); methodology (equal); project administration (equal); writing – original draft (supporting); writing – review and editing (equal).

## CONFLICT OF INTEREST

The authors have no financial or nonfinancial interests that are directly or indirectly related to the work submitted for publication.

## Supporting information


Video S1

## Data Availability

All data presented in this paper will be uploaded to Dryad upon paper acceptance.
